# Supplementary material for: Aptamer Trimode Biosensor for Trace Glyphosate Based on FeMOF Catalytic Oxidation of Tetramethylbenzidine
Source: Biosensors (Basel). 2022 Oct 25;12(11):920. doi: 10.3390/bios12110920 (PMC9688084; doi:10.3390/bios12110920)
Supplement: Supplementary file 1 [file biosensors-12-00920-s001.zip › biosensors-1965612-supplementary.pdf]

biosensors

MDPI

supplementary

Aptamer Trimode Biosensor for Trace Glyphosate Based on FeMOF Catalytic Oxidation of Tetramethylbenzidine

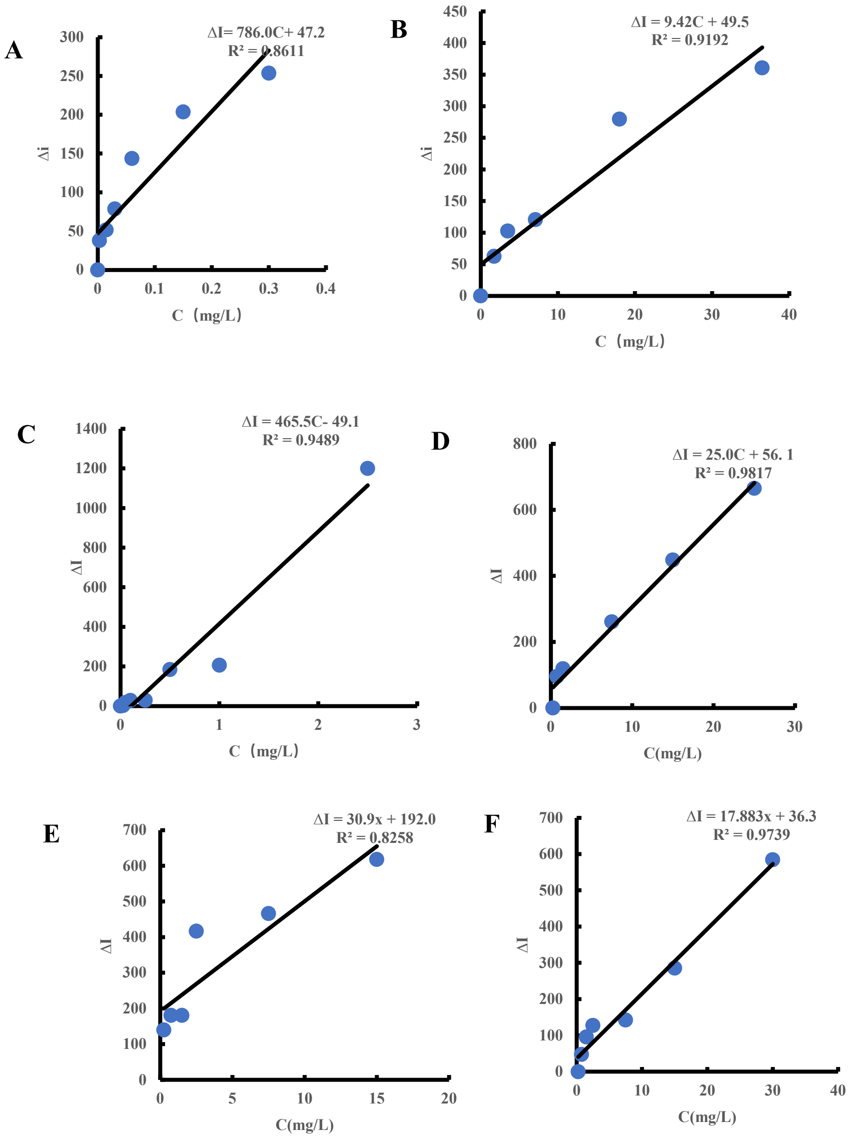

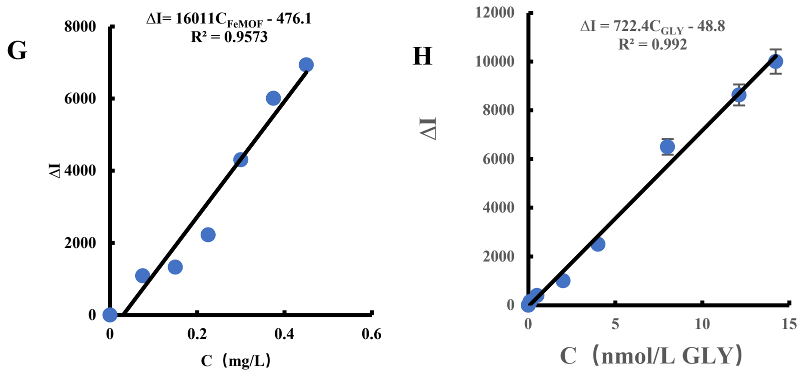

Figure S1. The relationship between SERS peak intensity and catalyst/GLY concentration. A: a: 0.45 mmol/L Tris-HCl + 4.5×10^{-6} mol/L H_2O_2 + 0.025 mmol/L TMB + 11.6 mg/L AgNPs；b： a+ 0.003 mg/L AuNPs；c： a+0.015 mg/L AuNPs；d： a+0.03mg/L AuNPs；e： a+0.06 mg/L AuNPs；f: a+0.15 mg/L AuNPs; g: a+0.3 mg/L AuNPs; **B:** a： 0.45 mmol/L Tris-HCl + 4.5×10^{-6} mol/L H_2O_2 + 0.025 mmol/L TMB + 11.6 mg/L AgNPs；b： a+ 1.725 mg/L AgNPs; c: a+3.5 mg/L AgNPs; d: a+7.1 mg/L AgNPs; e: a+18 mg/L AgNPs; f： a+36 mg/L AgNPs. **C:** a： 0.45 mmol/L Tris-HCl + 4.5×10^{-6} mol/L H_2O_2 + 0.025 mmol/L TMB + 11.6 mg/L AgNPs；b： a+ 0.025 g/L HPR; c: a+0.05 mg/L HPR; d： a+0.1 mg/L HPR；e： a+0.25 mg/L HPR；f： a+0.5 mg/L HPR; g: a+1 mg/L HPR; h: a+2.5 mg/L HPR. **D:** a: 0.45 mmol/L Tris-HCl + 4.5×10^{-6} mol/L H_2O_2 + 0.025 mol/L TMB + 11.6 mg/L AgNPs；b： a+ 0.25 mg/L CoMOF；c： a+0.75 mg/L CoMOF；d： a+1.5 mg/L CoMOF；e： a+7.5 mg/L CoMOF；f： a+0.015 g/L CoMOF；g： a+0.025g/L CoMOF. **E:** a: 0.45 mmol/L Tris-HCl + 4.5×10^{-6} mol/L H_2O_2 + 0.025 mol/L TMB + 11.6 mg/L AgNPs；b： a+ 0.25 mg/L CuMOF；c： a+0.75 mg/L CuMOF；d： a+1.5 mg/L CuMOF；e: a+2.5 mg/L CuMOF; f: a+7.5 mg/L CuMOF; g: a+0.015 g/L CuMOF; **F:** a: 0.45 mmol/L Tris-HCl + 4.5×10^{-6} mol/L H_2O_2 + 0.025 mol/L TMB + 11.6 mg/L AgNPs；b： a+ 0.25 mg/L NiMOF；c： a+0.75 mg/L NiMOF；d： a+1.5 mg/L NiMOF；e： a+2.5 mg/L NiMOF；f： a+7.5 mg/L NiMOF；g： a+0.015 g/L NiMOF；f： a+0.03 g/L NiMOF; **G:** a: 0.45 mmol/L Tris-HCl + 4.5×10^{-6} mol/L H_2O_2 + 0.025 mmol/L TMB + 11.6 mg/L AgNPs；b： a+ 0.25 mg/L FeMOF；c： a+0.75 mg/L FeMOF；d： a+1.5 mg/L FeMOF；e： a+2.5 mg/L FeMOF；f： a+7.5 mg/L FeMOF；g： a+0.015 g/L FeMOF；h： a+0.025g/L FeMOF；i： a+0.05g/L FeMOF; **H:** a: 0.45 mmol/L Tris-HCl + 4.5×10^{-6} mol/L H_2O_2 + 0.025 mmol/L TMB+7.5mg/L FeMOF + 0.01 μmol/L Apt_{GLY} + 11.6 mg/L AgNPs；b： a+ 0.1 nmol/L GLY；c： a+ 0.5 nmol/L GLY；d： a+2 nmol/L GLY；e： a+4 nmol/L GLY；f： a+8 μmol/L GLY；g： a+10 μmol/L GLY；h： a+14.22 nmol/L GLY.

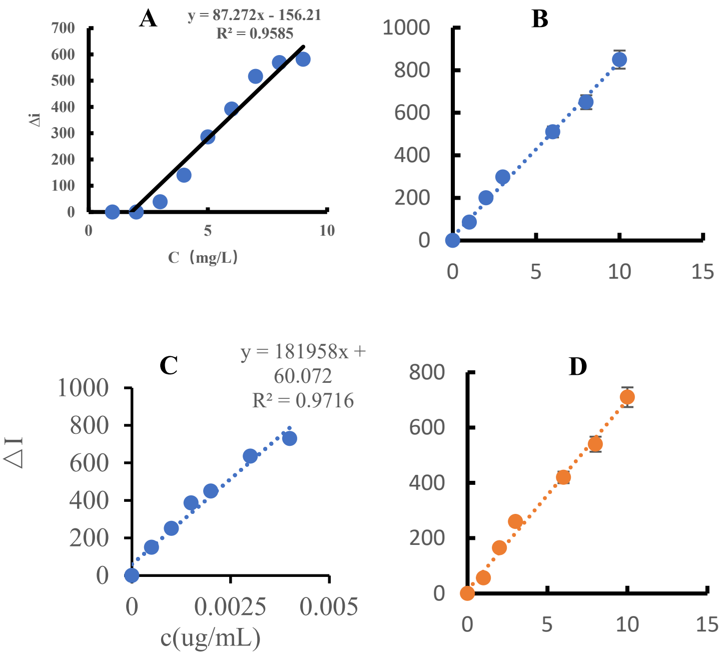

Figure S2. The relationship between RRS/fluorescence intensity and FeMOF/GLY. A：RRS, a：0.45 mmol/L Tris-HCl + 4.5×10^{-6} mol/L H_2O_2 + 0.025 mmol/L TMB + 11.6 mg/L AgNPs；b：a+ 0.25 mg/L FeMOF；c：a+0.75 mg/L FeMOF；d：a+1.5 mg/L FeMOF；e：a+2.5 mg/L FeMOF；f：a+7.5 mg/L FeMOF；g：a+0.015 g/L FeMOF；h：a+0.025g/L FeMOF；i：a+0.05g/L FeMOF；B: RRS, a：0.45 mmol/L Tris-HCl + 4.5×10^{-6} mol/L H_2O_2 + 0.025 mmol/L TMB+7.5mg/L FeMOF + 0.01 μmol/L Apt_{GLY} + 11.6 mg/L AgNPs；b：a+ 1 nmol/L GLY；c：a+2 nmol/L GLY；d：a+73 nmol/L GLY；e：a+6 nmol/L GLY；f：a+8 nmol/L GLY；g：a+10 nmol/L GLY. C: Fluorescence, with excitation wavelength of 300 nm, 2.5×10^{-4} mol/L TMB + 5×10^{-4} mol/L H_2O_2+ 0.45 mmol/L Tris-HCl + FeMOF (a~g: 0, 5×10^{-4}, 1×10^{-3}, 1.5×10^{-3}, 2×10^{-3}, 3×10^{-3}, 4×10^{-3} g/L FeMOF). F: Fluorescence, 2.5×10^{-4} mol/L TMB + 5×10^{-4} mol/L H_2O_2 +0.45 mmol/L Tris-HCl + 1 nmol/L Apt_{GLY}+xGLY (0, 1, 2, 3, 6, 8, 10 nmol/L GLY).

A B C

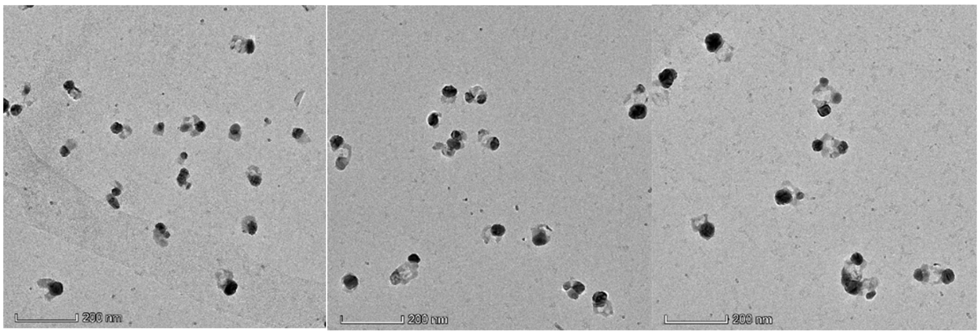

D

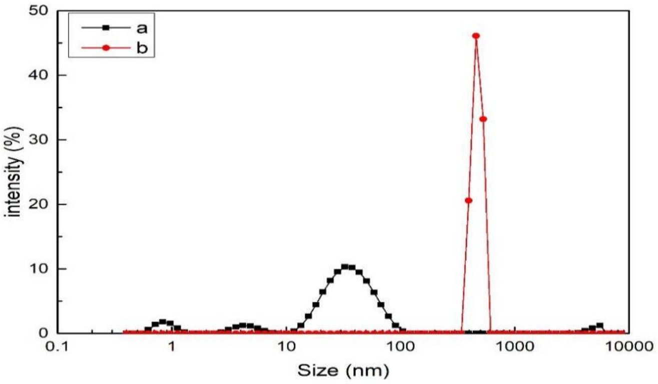

Figure S3. Transmission electron microscopy and laser scattering of FeMOF analytical system. A：4.0×10^{-4} mol/L AgNPs; B：0.45 mmol/L Tris-HCl + 4.5×10^{-6} mol/L H_2O_2 + 0.025 mmol/L TMB+7.5mg/L FeMOF + 0.01 μmol/L Apt_{GLY}+0.16 μmol/L AgNPs; C：0.45 mmol/L Tris-HCl + 4.5×10^{-6} mol/L H_2O_2 + 0.025 mmol/L TMB+7.5mg/L FeMOF + 0.01 μmol/L Apt_{GLY}+2.5 ng/L GLY+0.16 μmol/L AgNPs; D：Laser light scattering; a: Blank system; b: FeMOF analysis system.

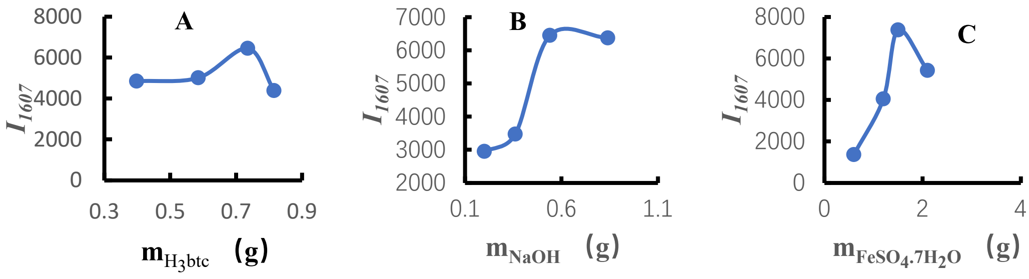

Figure. S4 Effect of preparation conditions on FeMOF.

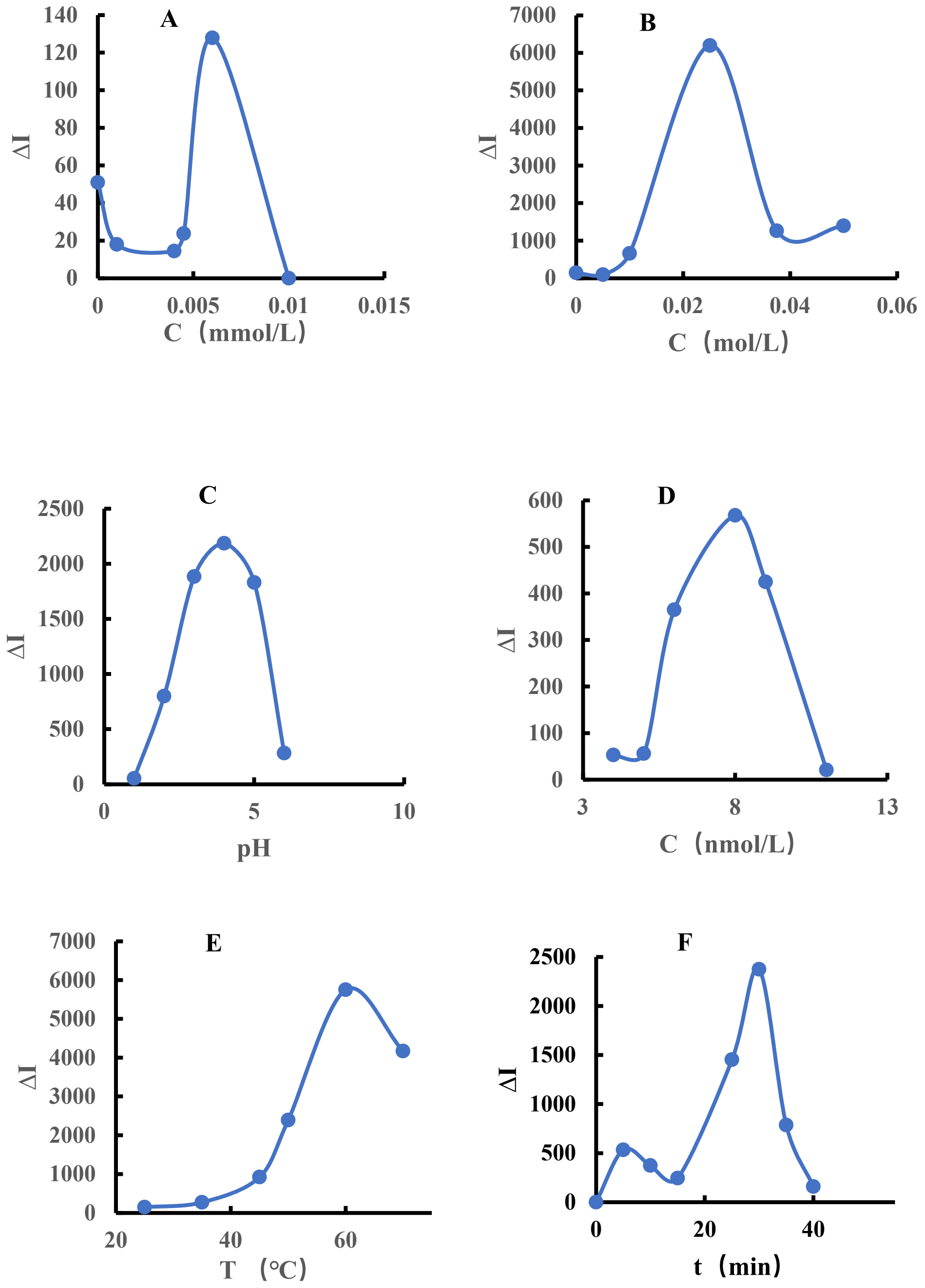

Figure S5. Influence of the analysis conditions. A: H_2O_2 concentration, 0.45 mmol/L Tris-HCl + 0.025 mmol/L TMB+7.5 mg/L FeMOF+0.1 μmol/L Apt_{GLY}+21.1 nmol/L GLY+ H_2O_2+ 11.6 mg/L AgNPs; B: TMB concentration, 0.45 mmol/L Tris-HCl + 4.5×10^{-6} mol/L H_2O_2 + 7.5 mg/L FeMOF+1.25 ng/L Apt_{GLY}+21.1 nmol/L GLY + TMB+ 11.6 mg/L AgNPs; C: Tris-HCl concentration, 0.1 μmol/L Apt_{Co} + 0.025 mmol/L TMB+7.5 mg/L FeMOF+ 4.5×10^{-6} mol/L Apt_{GLY}+21.1 nmol/L + Tris-HCl+ 11.6 mg/L AgNPs; D: Apt_{GLY} concentration, 0.45 mmol/L Tris-HCl + 0.025 mmol/L TMB+7.5 mg/L FeMOF+ 4.5×10^{-6} mol/L H_2O_2 +21.1 mol/L GLY+ Apt_{GLY}+ 11.6 mg/L AgNPs; E: Reaction time, 0.45 mmol/L Tris-HCl + 0.025 mmol/L TMB+7.5 mg/L FeMOF+0.1 μmol/L Apt_{Co}+0.1nmol /L $CoCl_2$+ 4.5×10^{-6} mol/L H_2O_2+ 11.6 mg/L AgNPs; F: Reaction temperature, 0.45 mmol/L Tris-HCl + 0.025 mmol/L TMB+7.5 mg/L FeMOF+0.1 μmol/L Apt_{Co}+0.1 nmol /L $CoCl_2$+ 4.5×10^{-6} mol/L H_2O_2+ 11.6 mg/L AgNPs.

Table S1. Effect of coexistence substance on the SERS determination of GLY.

**Coexisting substance**	**Relative multiples**	**Relative error（%）**	**Coexisting substance**	**Relative multiples**	**Relative error（%）**
PO_4^-	100	1.08	NH_4^+	100	−6.92
$P_2O_7^-$	50	5.6	NO_3^-	100	3.59
IO_3^-	100	3.29	Ba^{2+}	50	6.82
Br^-	100	−6.64	SiO_3^-	100	0.76
CO_3^{2-}	100	0.56	Al^{3+}	100	1.92
Mg^{2+}	100	−9.16	Fe^{3+}	50	−3.76
Sanmate	100	4.8	Phosphoramidothionate	100	3.9
Dipterex	100	5.6	BSA	100	4.2

Table S2. SERS results of GLY in wastewater samples.

**Sample**	**Mean value（μmol/L, n=5）**	**Added GLY（μmol/L）**	**Found (μmol/L)**	**Recovery（%）**	**RSD（%）**	**Content (μmol/L)**	**Ref results (μmol/L)**
1	0.382	0.169	0.385	93.6	3.6	0.764	0.750
2	0.226	0.169	0.537	92.1	8.7	0.0760	0.0712
3	0.338	0.169	0.527	97.5	6.3	0.0720	0.0686
4	0.196	0.169	0.356	94.6	5.4	0.0420	0.0401
5	0.113	0.169	0.275	96.1	4.8	0.0240	0.0280
